# COVID-19 Lockdown and Changes of the Dietary Pattern and Physical Activity Habits in a Cohort of Patients with Type 2 Diabetes Mellitus

**DOI:** 10.3390/nu12082327

**Published:** 2020-08-04

**Authors:** María Belén Ruiz-Roso, Carolina Knott-Torcal, Diana C. Matilla-Escalante, Alba Garcimartín, Miguel A. Sampedro-Nuñez, Alberto Dávalos, Mónica Marazuela

**Affiliations:** 1 Laboratory of Epigenetics of Lipid Metabolism, Madrid Institute for Advanced Studies (IMDEA)-Food, CEI UAM + CSIC, 28049 Madrid, Spain; belen.ruizroso@imdea.org (M.B.R.-R.); diana.mantilla@imdea.org (D.C.M.-E.); 2 Department of Endocrinology and Nutrition, Hospital Universitario de la Princesa, Instituto de Investigación Princesa, Universidad Autónoma de Madrid, 28049 Madrid, Spain; carolina.knott@salud.madrid.org (C.K.-T.); miguelantonio.sampedro@salud.madrid.org (M.A.S.-N.); 3 Departamento de Farmacología, Farmacognosia y Botánica, Facultad de Farmacia, Universidad Complutense de Madrid, 28040 Madrid, Spain; a.garcimartin@ucm.es

**Keywords:** COVID-19, lockdown, type 2 diabetes mellitus, diet, physical activity, food cravings

## Abstract

The COVID-19 lockdown clearly affected the lifestyle of the population and entailed changes in their daily habits, which involved potential health consequences, especially on patients with Type 2 Diabetes Mellitus (T2DM). We aimed to examine the impact of the lockdown caused by COVID-19 pandemic on both nutrition and exercise habits, as well as the psychological effects in patients with T2DM, compared to their usual diet and physical activity level previous to the complete home confinement. We also intended to analyse any potential variables that may have influenced these lifestyle modifications. A Food Frequency Questionnaire (FFQ), Physical Activity Questionnaire (IPAQ), Food Craving Questionnaire-State (FCQ-S) and Food Craving Questionnaire-Trait (FCQ-T) were used. Our results showed an increase in vegetable, sugary food and snack consumption. An association between levels of foods cravings and snack consumption was also found. Data also showed a high percentage of physical inactivity before the COVID-19 lockdown, which was exacerbated during the home confinement. These findings emphasise the great importance to do further research with larger study samples to analyse and explore dietary habits and to develop public health policies to promote a healthy lifestyle in terms of diet and physical activity in these patients, especially after this strict period of lockdown.

## 1. Introduction

A new form of coronavirus (SARS-CoV-2) emerged, alerting the medical and scientific communities on December 2019 [1]. The resulting disease has been named as COVID-19 and considered a global health emergency by The World Health Organisation (WHO). Moreover, the current global pandemic has led to a great number of infections and more than 570.288 deaths according to the official statistics worldwide [2].

One of the measures that have been adopted by the governments from different countries worldwide, especially those more affected by the pandemic, was full lockdown of the cities. This situation has led to a disruption of the daily activities of the population [3]. In order to contain the spread of the virus and to avoid the health system to collapse, the Spanish government released on the 14th of March an executive order to implement a state of alarm, when strict lockdown measures such as social distancing and complete confinement at home were first imposed [4].

Lockdown can clearly impact the lifestyle of the population, especially in terms of diet and physical activity. Many scientific organisations, such as the WHO and The Spanish Academy of Nutrition and Dietetics [5], have acknowledged the crucial role of physical activity and nutrition in both the prevention and treatment of non-transmissible chronic diseases [6]. These organisations have published recommendations related to food and nutrition during the period of lockdown, since there is a close relationship between the quality of the diet and the overall health state [7], especially in patients with type 2 diabetes mellitus (T2DM). In T2DM, exercise and diet play an important role in the management of the disease, and any disruptive changes may result in major health effects and a worsening in the metabolic control of their diabetes [8].

During the COVID-19 outbreak, patients were advised to avoid going to hospitals unless strictly necessary [9]. Therefore, management of T2DM may have been more difficult and often based on virtual consultations. Furthermore, the lockdown caused by COVID-19 pandemic has also increased feelings of stress or anxiety in these patients since they have been considered a high-risk group according to health authorities [10]. Additionally, it is important to highlight that patient education and self-care promotion are key aspects in the proper management of their disease [11]. In the context of the aforementioned situation, we believe that it is important to assess the impact of the lockdown in the quality of the diet of patients with T2DM as well as the physical activity levels and their potential influence in this disease.

To this end, we aimed to examine the impact of the lockdown caused by COVID-19 pandemic on both nutrition and exercise habits, as well as the psychological effects in patients with T2DM. We compare this to their usual diet and physical activity level before the complete home confinement. We also intended to analyse any potential variables that may have influenced these lifestyle modifications.

## 2. Material and Methods

### 2.1. Participants

The population chosen for our study was a cohort of adult patients with T2DM from our site, University Hospital La Princesa (Madrid, Spain), with different progression of the disease and treatments. In order to participate in the study, all the participants previously agreed to give oral consent. The inclusion criteria to participate in the study were: (1) male or female between the age of 40 and 80, (2) previous diagnosis of T2DM, (3) BMI ≥25 and <40 kg/m^2^. The vast majority of our patients presented other comorbidities such as dyslipidaemia. The inclusion criteria were selected in order to get a homogeneous study population and to avoid any potential confounding factors.

### 2.2. Study Design

This cross-sectional study was conducted between the 8th of April 2020 and the 20th of May 2020. We collected data from patients with T2DM through phone interviews regarding dietary habits, physical activity and food craving. The interviews were conducted by two investigators (Nutritionists) from University Hospital La Princesa (Madrid, Spain) and the IMDEA Food Institute (Madrid, Spain). Each interview lasted approximately 30–45 min according to patients’ response.

Data were collected through a questionnaire divided into different realms. Standardised questionnaires were used to evaluate all the variables. Dietary practices were evaluated using a semi-quantitative 96-item questionnaire called Food Frequency Questionnaire (FFQ), validated in Spanish adults [12,13]. Each item included a typical portion size. For each food item, weekly food intake was estimated by multiplying the portion size by the frequency of consumption per week. Then, we grouped each item in ten food groups (Sugary food, Sugar-sweetened beverage, snacks, nuts, cereals, legumes, dairy products, vegetables, fruits and meat, fish and eggs).

To assess the level of physical activity, we use the International Physical Activity Questionnaire (IPAQ) [14], which allows to classify the level of physical activity by time spent doing moderate physical activity and walking as well as time spent sitting per week. The participants of the study reported the servings consumed of all the different food groups and time spent on physical activity during the week before lockdown and one week during lockdown. Additionally, food craving was evaluated using the validated Food Craving Questionnaire-State (FCQ-S) and Food Craving Questionnaire-Trait (FCQ-T) during the COVID-19 lockdown [15].

Demographic, biological, and anthropometric data, which included gender, age, capillary glycated haemoglobin (HbA1c) values and BMI, were collected from the patients’ medical records. These data were gathered by the nurse, the endocrinologist or the nutritionist from University Hospital La Princesa (Madrid, Spain), in their last medical consultation within two months prior to the COVID-19 lockdown.

The adherence to dietary guidelines and recommendations of healthy diet during the COVID-19 lockdown [7] have been assessed in our patients using a network map and have been categorised by the general characteristics of the patients. In this context, patients were classified according to whether they comply with the recommended intake for each food group. Patients were also classified in two groups using the average age (44 to 63 and 64 to 77). The BMI was calculated as the ratio of body weight in kilograms divided by the height in squared metres (kg/m^2^). Following this, the participants were classified in groups: (1) 25.0 to <30, (2) 30 to <35 and 35 to <40. Capillary HbA1c values are indicated as the percentage of glycated haemoglobin, and patients were divided as %HbA1c < 6.5% (optimal control) or %HbA1c ≥ 6.5% (sub-optimal control). A network map of coincidences was built using these data.

### 2.3. Data Analysis

Initially, the average intake of different food groups amongst the participants was compared both before and during the COVID-19 lockdown using a paired two-tail Student’s *t*-test. Two-way ANOVA was used to evaluate the effect of gender, age, body mass index (BMI) and capillary HbA1c on the difference of average weekly intake and exercise data, both before and during the COVID-19 lockdown (interaction *p*-value). The independent categorical variables were assessed using the Pearson correlation coefficient. A 95% confidence interval (95% CI) was selected, and a significance level of *p* < 0.05 was applied to all statistical analyses. Corrplot R package was used for correlation map graphics. Finally, netCoin R package [16] was used to create a network graph of coincidences within the different group of patients during the COVID-19 lockdown. Stata (v. 12.0, College Station, TX, USA) GraphPad Prism 8 (version 8.3.0; Graph Pad Software Inc. San Diego, CA, USA) and R version 3.5.1 were used for all statistical analyses.

### 2.4. Ethics Approval and Consent to Participate

Ethical approval for this project was granted by the Internal Ethical Review Committee of the University Hospital de La Princesa (Registration number: 4086) and oral informed consent was given by all the participants prior to their inclusion, in accordance with the Declaration of Helsinki.

## 3. Results and Discussion

A total of 102 patients initially met the eligibility criteria. Amongst those, 30 (30.6%) declined to participate. The final sample consisted of 72 patients with T2DM aged 45–77 years. The average age was 63 years and the sample was divided between males (48.6%) and females (51.4%). All general characteristics of the study sample are included in Table 1.

### 3.1. Changes in Dietary Patterns of Patients with Type 2 Diabetes during the COVID-19 Lockdown

**Figure 1:**
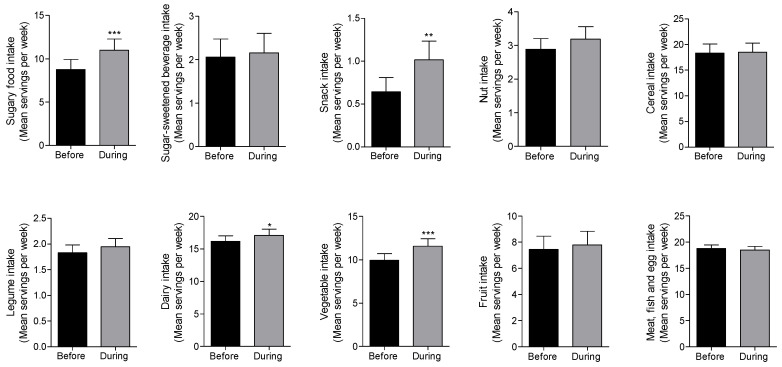
Comparison of mean dietary intake among patients with Type 2 Diabetes before and during the COVID-19 lockdown. Data is shown as means ± SEM. Comparison between groups were performed using a paired two-tail Student’s *t*-test. * *p* < 0.005, ** *p* < 0.001, *** *p* < 0.0001. *n* = 72.

**Figure 2:**
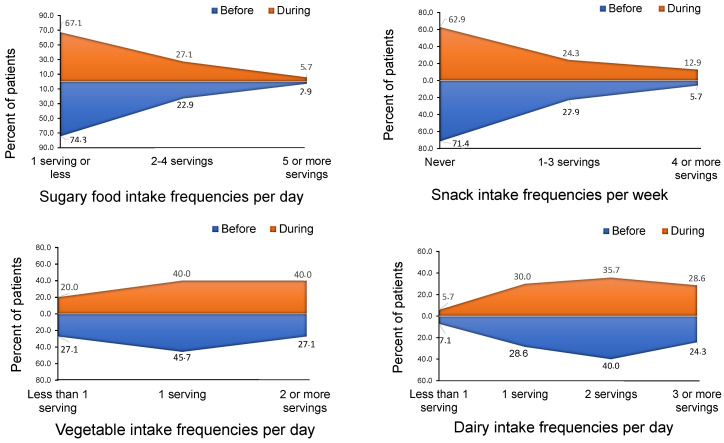
Food intake frequency to compare dietary patterns before and during the COVID-19 lockdown, expressed by percentage of patients according to their weekly or daily frequency of consumption of each food group. *n* = 72.

Figure 1 shows the average intake of the food groups per week before and during the COVID-19 lockdown. The intake of dairy products, vegetables, snacks and sugary foods significantly increased during lockdown in these patients, with both sugary foods and snacks as the most notable ones (Figure 1). Figure 2 shows the food intake frequency to compare dietary patterns before and during COVID-19 lockdown, expressed by the percentage of patients according to their daily or weekly frequency of consumption of sugary foods, snacks, vegetables and dairy products as they were the most relevant results. Supplementary Table S1 includes the rest of the food groups (fruit, meat, fish and eggs, cereal, nut, legumes and sugar-sweetened beverages). 

The percentage of patients eating 1, 2–4 and 5 or more serving per week of sugary foods as well as the percentage of those eating snacks fewer than 1, 1–3 and 4 or more servings per week changed during lockdown. Whereas 74.3% did not used to have any sugary foods at all before lockdown, during lockdown that percentage decreased to 67.1%. Additionally, before lockdown just 2.9% used to eat snacks 5 or more times per week, increasing to 5.7% during lockdown. Likewise, the number of patients who ate this food group between 2 to 4 times per week also increased during that period (Figure 2). Furthermore, 71.1% of our patients did not used to have snacks before lockdown, which decreased to 62.9% during lockdown. Similarly, the percentage of patients that ate snacks between 1 to 3 times per week also increased during lockdown. It is noteworthy that whereas before lockdown just 5.7% consumed ≥ 4 times/week this food group, during lockdown it increased to 12.9% amongst the patients who participated in our study.

Several studies have previously linked an excessive consumption of sugary foods and snacking with environmental, cognitive and affective variables, as well as emotions such as boredom and stressful situations [17]. Although the psychological consequences of this pandemic remain unclear to date, experts believe that these will be significant and substantial [18]. It is very likely that the situation derived from the COVID-19 pandemic has unleashed the acquisition of bad dietary habits, thus increasing the consumption of sugary foods and snacks, which in these patients could involve a worsening of the metabolic control of their T2DM.

It is also important to highlight the changes in the consumption pattern of vegetables in our study population towards a positive tendency. In addition to the significant increase in the average vegetable consumption during lockdown (Figure 1), we further observed a decrease in the percentage of patients eating this food group ≤1 time per week as well as an increase in those eating vegetables ≥2 times per week, from 27.1% to 40% (Figure 2). Previous studies from our group also reported an increased vegetable intake during the COVID-19 lockdown amongst families from different countries of Europe and America [19]. These results are consistent with the data published by The Centre for the Promotion of Imports from developing countries, as they pointed out an increase in the overall vegetable sales, including fresh, tinned and frozen ones [20]. This could be explained, in part, because families would have had more time to prepare more elaborate home-cooked meals.

Although recent studies have not found a clear association between vegetable consumption and a lower risk to have T2DM [21], some authors claim that there is a relationship between fruit and vegetable consumption and a lower incidence of some chronic conditions such as coronary heart disease, cancer, T2DM or neurodegenerative diseases [22]. Furthermore, according to WHO legumes, fruits and vegetables are considered high-quality food groups and their consumption should be prioritised during the lockdown period or long stays indoors [23].

Regarding frequency of consumption of dairy products, we observed a decrease in the percentage of patients with T2DM that consumed one or fewer products per day from 7.1% before lockdown to 5.7% during this period. Likewise, the percentage of patients consuming 3 or more dairy products per day increased from 24.3% initially to 28.6% during lockdown (Figure 2).

In relation to this, some recent studies have yielded evidence about the inverse association between dairy products consumption and risk of T2DM [24]. However, it is important to note that this benefit derived from dairy products it is mainly due to the intake of low-fat fermented products [25], and that the recommendations for patients with diabetes are 3–4 portions of dairy foods per day [26].

There were no modifications in the consumption of sugar-sweetened beverages (SSB), nuts, cereals, legumes and fruits, as well as meat, fish and eggs in these patients during lockdown (Figure 1).

### 3.2. Changes of Dietary Habits of Patients with Type 2 Diabetes Mellitus According to Their Sociodemographic Characteristics before and during Lockdown

**Figure 3:**
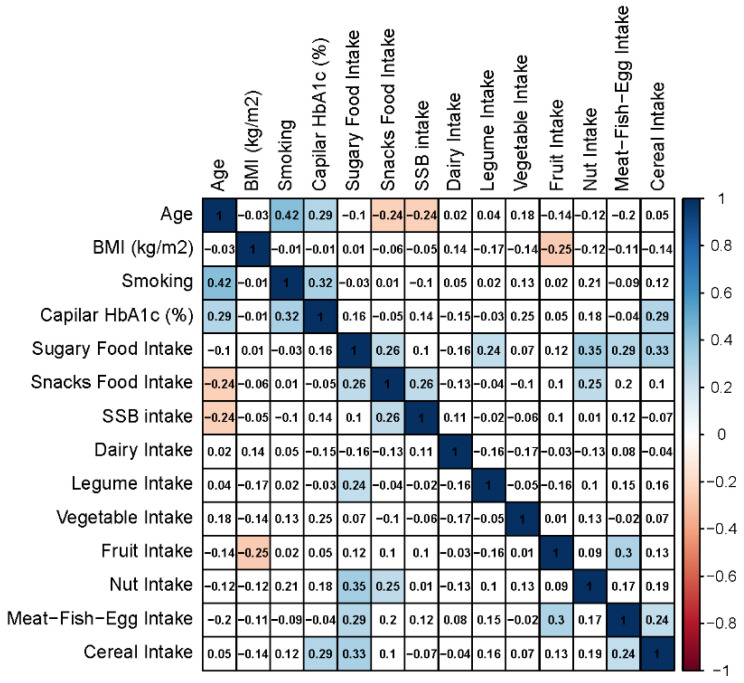
Correlation matrix for dietary patterns and general characteristics of the sample during the COVID-19 lockdown. Red and blue cells indicate significant indirect and direct correlations (*p* < 0.05), respectively. White squares show non-significant correlations (*p* > 0.05), numbers inside cells indicate Pearson correlation coefficient (*r*).

**Figure 4:**
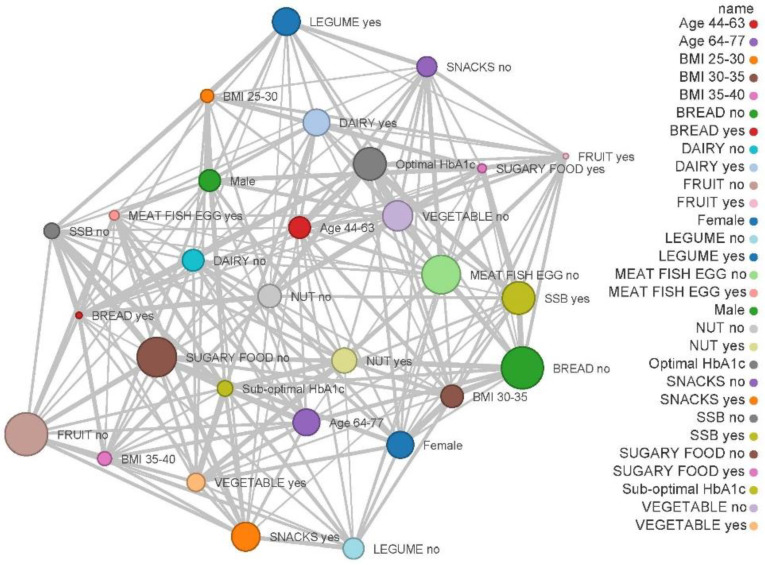
Networking mapping of type 2 diabetes mellitus (T2DM) patients that maintained (yes) or did not maintain (no) a suitable food intake according to the dietary guidelines by general characteristics of the sample during the COVID-19 lockdown. Circle size correlate to the prevalence of that variable and a shorter distance between the circles indicates a greater co-occurrence of features.

Table 2 shows the changes in dietary patterns due to the COVID-19 lockdown in patients with T2DM who agreed to participate in the survey according to different variables such as gender, age, BMI and HbA1c. Figure 3 shows the correlation between dietary patterns and general characteristics of the sample during lockdown. Figure 4 represents network graph regarding adherence to dietary guidelines and healthy diet recommendations, classified by the general characteristics of the patients, during the COVID-19 lockdown. Only the most remarkable results will be discussed.

### 3.3. Gender

The gender classification results show that females significantly increased their sugary food (from 7.2 before to 9.4 servings per week during lockdown, *p* = 0.0036), and snack intake (from 0.7 to 1.3 servings per week during lockdown, *p* = 0.0025) as well as vegetable intake (from 10.7 before to 12.2 servings per week during lockdown, *p*= 0.001) during lockdown (Table 2). On the other hand, males also showed an increase in sugary foods (from 10.1 to 12.3 servings per week, *p* = 0.0013) and vegetable consumption (from 9.1 before to 10.8 servings per week during the COVID-19 lockdown, *p* = 0.0049), but did not change their average snack consumption (Table 2). These results are consistent with previous observational studies about dietary patterns and gender [27], which have shown differences between the genders regarding eating behaviour and food choices, especially in sugary food consumption. It is important to highlight these data since sugary foods are linked to overweight and obesity, T2DM and cardiovascular disease, amongst other detrimental health effects [28].

Interestingly, cereal intake followed a different tendency in both genders (interaction *p* = 0.049). While males increased their weekly intake from 17.7 to 19.7, females decreased theirs from 18.6 to 17.0. (Table 2).

### 3.4. Age

Age is considered an important risk factor for T2DM and the burden of the disease is very high in older age groups [29]. In our study, patients aged 63 years or younger significantly increased sugary food intake (from 10.2 to 14.2 servings per week, *p* < 0.0001) as well as snacks (0.9 to 1.5 servings per week, *p* = 0.0037) during the COVID-19 lockdown, unlike patients aged over 64 years old, who did not change the consumption of sugary foods or snacks during that period (Table 2). This could be explained by the fact that older patients have greater awareness of the importance of dietary habits on the metabolic control of their disease. However, age only had a significant impact in sugary food consumption (interaction *p* = 0.003) (Table 2). At the same time, we found an inverse correlation between the age and the consumption of snacks and SSB during lockdown (Figure 3). These results should be taken into account to individualise management plans, especially in older people, to have stricter control and focus on the quality of life of these patients and their potential comorbidities due to the diet.

### 3.5. Body-Mass Index (BMI)

Our results showed that BMI significantly influenced the change in nuts consumption (interaction *p* = 0.0032) as well as meat, fish and eggs intake due to the COVID-19 lockdown (interaction *p* = 0.0022) (Table 2). On the other hand, we noted that there was an inverse correlation between the weekly consumption of fruit servings and the BMI (Figure 3). We further observed that those patients with a BMI between 35 to 40 kg/m^2^ significantly increased sugary food consumption (*p* = 0.001), as opposed to the group with the BMI between 30 and 35 kg/m^2^, who consumed the lowest number of servings of sugary foods during lockdown (Table 2). Figure 4 shows that the highest rates of adherence to the weekly recommended sugary food and snack consumption were in those patients with a lower BMI. Similarly, our results are consistent with previous research [30,31], as we also found this relationship between quality of the diet and BMI.

### 3.6. Capillary Glycated Haemoglobin (HbA1c)

Patients with HbA1c values lower than 6.5% increased their sugary food and snack intake (from 7.8 to 10.3 servings per week, *p* < 0.0001; from 0.7 to 1.1, *p* = 0.0046, respectively), and dairy products consumption (from 16.3 to 17.8 servings per week, *p* = 0.0342) during the COVID-19 lockdown. However, those with HbA1c values ≥ 6.5% showed a higher consumption of sugary foods than the other group, both before and during the COVID-19 lockdown (Table 2). However, the group of patients with HbA1c ≥6.5% showed a higher consumption of sugary foods than the other group before and during the COVID-19 lockdown (Table 2). HbA1c did not influence the change in the intake of these food groups due to the COVID-19 lockdown (Table 2). However, it is important to highlight that HbA1c significantly influenced the change of SSB consumption (interaction *p* = 0.009). The group with HbA1c values ≥6.5% showed a higher average SSB intake during the COVID-19 lockdown (2.9 servings per week) compared to the group with HbA1c values <6.5% (1.5 servings per week) (Table 2). Several observational studies and meta-analyses have shown that a high intake of SSB is associated with the development of T2DM [32].

Additionally, we found a positive correlation between capillary HbA1c and cereal intake (Figure 3). These findings have been demonstrated by Haimoto et al. [32], who found a positive correlation between carbohydrate intake and a higher percentage of capillary HbA1c.

Figure 4 shows that the highest rates of adherence to the recommended daily or weekly servings of each food group were in those patients with better control of their HbA1c.

### 3.7. Changes in Physical Activity Habits during the COVID-19 Lockdown of Patients with T2DM

**Figure 5:**
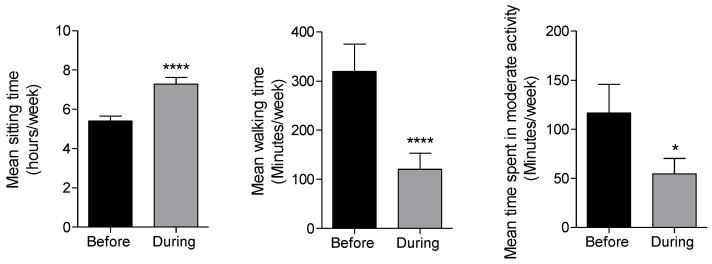
Comparison of mean sitting, walking and moderate physical activity time per week before lockdown and during lockdown among patients with Type II Diabetes. Data are shown as means ± SEM. Comparison between groups by paired two-tail Student’s *t*-test. * *p* < 0.05, **** *p* < 0.0001. *n* = 72.

During the COVID-19 lockdown, we noticed a significant increase in the daily hours that the participants of the study were sitting without doing any physical activity at all (Figure 5). Regarding the average minutes per week spent walking, we observed a significant decrease during lockdown compared to the period before. Additionally, patients reported a decline in the average weekly time spent doing any type of moderate physical activity (Figure 5). This is not an unexpected finding, since one of the main containment measures to restrain the COVID-19 spread was total home confinement. On the other hand, these results also showed a high percentage of our study sample with a low physical activity level both before and during lockdown. A multitude of studies have found a relationship between being physically active and a lower risk to develop T2DM, partly mediated by a reduced adiposity [33]. Furthermore, physical activity is directly associated with a HbA1c reduction in patients with T2DM when combined with a healthy diet [34].

Apart from the aforementioned metabolic improvements, physical activity is known to have a positive impact in the mental health realm [35]. This has been particularly relevant during this period, since mental health may have been affected during this pandemic by the measures of social distancing. Therefore, identifying changes in the physical activity levels during the COVID-19 lockdown may represent an important contribution to studies about health factors in patients with T2DM, as insufficient physical activity levels are considered one of the main risk factors of cardiovascular disease, cancer, T2DM as well as deaths worldwide (OMS, 2018).

When comparing physical activity levels from both periods (before and during lockdown) according to the general variables of our patients, it was shown that both men and women followed the same pattern, which means that they significantly increased their time of inactivity during lockdown compared to before this situation (Table 3). It is important to highlight lower physical activity levels in women with T2DM before lockdown than in men. However, it is not clear to date which specific factors might explain these differences between genders [36]. Conversely, patients with T2DM who were ≥ 64 years old did not present remarkable changes in moderate physical activity levels, since previously to lockdown they did not spend much time in this type of activities either. However, both groups of age significantly increased their time of inactivity (Table 3).

Apart from that, patients with T2DM with a BMI > 30 kg/m^2^ showed a significant increment in the hours they spent sitting, whereas those with a BMI between 25 to 30 kg/m^2^ did not increase these hours (Table 3). Patients with a HbA1c lower than 6.5% used to spend more time doing moderate physical activity before lockdown, although it declined during this time due to the home confinement. Nevertheless, those with a HbA1c higher than 6.5% did not have any changes in their moderate physical activity level (Table 3).

### 3.8. Relationship between Food Cravings due to the COVID-19 Lockdown and Sociodemographic, Dietary and Physical Activity Characteristics

It is known that dietary choices are strongly influenced by psychological factors, in which lockdown had an evident impact too [37]. Therefore, all these potential lifestyle changes brought about by the lockdown are worth investigating as their long-term health effects are still unknown and of great interest for the scientific organisations. Different factors related to mental health such as stress, social isolation or lack of physical activity can play a role in the onset of food cravings, which might be quite frequent in patients with T2DM [38], causing them to snack more frequently throughout the day. To assess the food cravings in this cohort of patients and how they were associated with the other variables, we used the validated questionnaires FCQ-T and FCQ-S. The results from this analysis suggested that there was a significant correlation between anxiety levels and the gender, BMI and HbA1c values during the COVID-19 lockdown (Table 4). More specifically, women reported more food cravings than men, and patients with a higher BMI also reported more food cravings. On the contrary, age did not show any influence in food cravings amongst these patients.

We have to emphasise that the increase in snacks, vegetables and dairy products consumption was significantly related to levels of food cravings in our cohort of patients during the lockdown (Table 4). Furthermore, we also found a correlation between fruit intake and food cravings (Table 4). This could be explained by the fact that being completely confined at home contributed to an increase of food cravings, which translated into a higher consumption of products such as snacks and dairy. However, we could not find any correlation between food cravings and consumption of sugary foods (Table 4). Data from a meta-analysis study with a sample of 3292 participants demonstrated that those patients with a higher BMI and weight had more food cravings. Additionally, overweight subjects with pharmacological treatments to reduce anxiety increased weight loss [39]. It is likely that the COVID-19 lockdown, apart from triggering emotions such as boredom or anxiety, unleashed the appearance of food cravings and had an impact on weight in the short term, which could worsen the metabolic control of their T2DM in the long term.

The severe lockdown measures, such as social distancing and school closures, as well as restrictions on group gatherings and physical activities in open spaces and dedicated facilities, abruptly turned upside down the traditional lifestyle. All of this has had consequences on the psychological and emotional states of individuals. A strength of our study was that the survey was conducted during the state of alarm, which was the most critical period, as the country was in full lockdown. As limitations of the study, it should be noted that patients’ weight and caloric intake were not assessed. Moreover, 30.6% of patients previously selected declined to participate. Furthermore, as the interviews were carried out by phone, there is a response bias that could have influenced the answers of the study participants. This may include fatigue from answering so many questions or difficulty to recall dietary and physical activity habits before the COVID-19 lockdown.

## 4. Conclusions

In conclusion, our findings provide the first description of how the COVID-19 lockdown has modified dietary patterns and physical activity habits in patients from Spain with T2DM, as well as its relationship with food cravings during this period. These new habits could be further maintained, which would have a negative impact in the metabolic control of their T2DM and their health. It seems that during lockdown, families tried to ameliorate their dietary habits, as for example, they had more time to cook more elaborate meals and increased their vegetable consumption, but despite this, the overall quality of the diet did not improve. They also showed an increase in sugary foods and snacks intake, probably due to emotions such as boredom of staying at home all day or the stress caused by the pandemic. In fact, we found an association between levels of foods cravings and snack consumption. Apart from that, this study found a high percentage of physical inactivity before the COVID-19 lockdown, which was exacerbated during home confinement. These findings emphasise the great importance to do further research with larger study samples to analyse and explore dietary habits and to develop public health policies to promote a healthy lifestyle in terms of diet and physical activity, especially after this strict period of lockdown. Because T2DM is a major burden on our health system, understanding the dietary pattern and physical activity habits during the lockdown in this specific population will help the national health authorities shape their response to future pandemics or other unavoidable global disasters where lockdown measures would be encouraged.

## Figures and Tables

**Table 1:** General characteristics of the 72 patients who filled out the questionnaire.

Characteristics	Sample (%)
Gender (*n* = 72)	
Male	48.6
Female	51.4
Age (years) (*n* = 72)	
44 to 63	48.6
64 to 77	51.4
Body-mass-index (kg/m^2^) (*n* = 63)	
25.0 to <30	16.7
30 to <35	63.0
35 to <40	20.4
Capillary HbA1c (*n* = 59)	
<6.5%	69.5
≥6.5%	30.5

**Table 2:** Comparison of patients’ dietary patterns before and during the COVID-19 lockdown, classified by gender, age, body mass index and capillary HbA1c (%).

		Gender (*n* = 72)		Age (Years) (*n* = 72)		Body-Mass-Index (kg/m^2^) (*n* = 63)			Capillary HbA1c (*n* = 59)	
		Male	Female	44 to 63	64 to 77	25.0 to <30	30 to <35	35 to <40	<6.5%	≥6.5%
Sugary food	Mean (95% CI) before lockdown	10.1 (6.1–14.1)	7.2 (4.6–9.7)	10.2 (6.1–14.3)	8.3 (5.2–11.4)	11.7 (4.2–19.1)	7.9 (4.3–11.5)	9.3 (5.2–13.3)	7.8 (5.1–10.5)	13.1 (6.4–19.8)
	Mean (95% CI) during lockdown	12.3 (7.6–16.9)	9.4 (6.8–12.0)	14.2 (9.3–19.0)	9.2 (6.3–12.1)	13.9 (5.0–22.8)	9.7 (6.2–13.2)	12.9 (8.3–17.5)	10.3 (7.1–13.6)	14.7 (7.8–21.6)
	*p*-value	0.0013 **	0.0036 **	<0.0001 ****	0.1035	0.0649	0.0307 *	0.001 ***	<0.0001 ****	0.1337
	Interaction *p*-value	0.96		0.003 **		0.69			0.4	
Snacks	Mean (95% CI) before lockdown	0.57 (0.1–1.1)	0.7 (0.3–1.2)	0.9 (0.5–1.5)	0.3 (0.1–0.5)	0.4 (−0.1–0.8)	0.9 (0.3–1.5)	0.3 (−0.04–0.6)	0.7 (0.3–1.1)	0.3 (−0.1–0.5)
	Mean (95% CI) during lockdown	0.7 (0.2–1.3)	1.3 (0.6–1.9)	1.5 (0.7–2.4)	0.5 (0.2–0.8)	0.7 (0.1–1.5)	1.3 (0.5–2.1)	0.7 (−0.02–1.3)	1.1 (0.5–1.6)	0.6 (−0.1–1.3)
	*p*-value	0.07	0.0025 **	0.0037 **	0.0326	0.1362	0.0133 *	0.0896	0.0046**	0.1106
	Interaction *p*-value	0.097		0.078		0.85			0.94	
Sugar-sweetened beverages (SSB)	Mean (95% CI) before lockdown	2.3 (1.0–3.6)	1.8 (0.7–2.8)	2.6 (1.1–4.1)	1.2 (0.4–2.0)	1.9 (0.5–3.3)	2.4 (0.7–4.0)	1.3 (0.2–2.3)	1.6 (0.5–2.7)	2.2 (0.7–3.7)
	Mean (95% CI) during lockdown	2.6 (1.1–4.1)	1.7 (0.6–2.7)	2.6 (1.1–4.1)	1.4 (0.3–2.5)	2.2 (0.1–4.3)	2.5 (0.8–4.2)	1.2 (0.1–2.3)	1.5 (0.4–2.5)	2.9 (1.0–4.9).
	*p*-value	0.2985	0.3242	>0.9999	0.4452	0.6165	0.3265	0.8474	0.1643	0.105
	Interaction *p*-value	0.19		0.49		0.47			0.009 **	
Diary	Mean (95% CI) before lockdown	18.3 (14.7–20.8)	14.5 (12.2–16.8)	15.7 (13.3–18.2)	15.5 (13.2–17.9)	14.3 (11.6–17.1)	16.0 (13.2–18.8)	16.9 (13.6–20.2)	16.3 (14.0–18.5)	15.2 (12.5–18.0)
	Mean (95% CI) during lockdown	19.0 (16.29–21.7)	15.5 (12.9–18.2)	16.3 (16.6–18.9)	16.9 (14.1–19.8)	14.5 (11.9–17.2)	17.5 (14.1–20.8)	17.8 (14.0–24.6)	17.5 (14.8–20.2)	15.7 (13.1–18.2)
	*p*-value	0.0871	0.0514	0.1431	0.0335	0.486	0.0275 *	0.2513	0.0342 *	0.2698
	Interaction *p*-value	0.7		0.26		0.4			0.38	
Legume	Mean (95% CI) before lockdown	2.0 (1.5–2.4)	1.7 (1.3–2.1)	1.8 (1.4–2.3)	1.8 (1.3–2.3)	2.1 (1.5–2.7)	1.7 (1.3–2.1)	1.9 (1.0–2.7)	1.9 (1.5–2.4)	1.7 (1.1–2.3)
	Mean (95% CI) during lockdown	2.1 (1.5–2.4)	1.8 (1.4–2.3)	1.8 (1.3–2.3)	2.0 (1.5–2.5)	2.4 (1.6–3.2)	1.7 (1.3–2.1)	2.0 (1.1–2.8)	2.0 (1.6–2.5)	1.9 (1.2–2.5)
	*p*-value	0.1835 (1.6–2.6)	0.1687	0.6621	0.0506	0.0961	0.6632	0.3299	0.2099	0.1631
	Interaction *p*-value	0.65		0.16		0.25			0.43	
Vegetable	Mean (95% CI) before lockdown	9.1 (6.7–11.4)	10.7 (8.7–12.8)	9.1 (6.9–11.3)	11.5 (9.1–14.0)	13.2 (9.4–17.0)	8.9 (6.6–11.2)	10.3 (6.9–13.6)	9.2 (7.2–11.2)	12.7 (8.9–16.4)
	Mean (95% CI) during lockdown	10.8 (8.1–13.5)	12.2 (9.9–14.4)	10.8 (8.2–13.4)	13.3 (10.7–16.0)	14.6 (9.6–19.6)	10.7 (8.1–13.3)	12.4 (9.1–15.6)	10.6 (8.4–12.8)	15.4 (11.3–19.5)
	*p*-value	0.0049 **	0.001 **	0.003 **	0.0033 **	0.0707	0.0018 **	0.0279 *	0.0071 **	0.0029 **
	Interaction *p*-value	0.7		0.74		0.7			0.14	
Fruit	Mean (95% CI) before lockdown	7.7 (4.8–5.4)	7.4 (4.6–10.2)	9.1 (5.7–12.6)	6.9 (4.2–9.7)	10.7 (4.5–17.0)	8.3 (5.1–11.6)	5.4 (2.2–8.5)	7.5 (4.6–10.3)	9.5 (4.9–14.1)
	Mean (95% CI) during lockdown	8.5 (10.7–11.6)	7.5 (4.6–10.39	9.7 (6.2–13.2)	7.2 (4.4–9.9)	11.4 (5.3–17.5)	8.9 (5.6–12.3)	5.3 (2.1–8.4)	7.7 (4.7–10.6)	10.2 (5.7–14.6)
	*p*-value	0.0133 *	0.744	0.0608	0.4508	0.1264	0.0577	0.7894	0.4484	0.0617
	Interaction *p*-value	0.075		0.81		0.42			0.3	
Nut	Mean (95% CI) before lockdown	2.9 (1.9–3.8)	2.8 (2.0–3.7)	3.4 (2.4–4.3)	2.6 (1.7–3.5)	2.2 (0.9–3.5)	3.6 (2.6–4.7)	2.7 (1.3–4.0)	2.9 (2.0–3.8)	3.1 (1.8–4.4)
	Mean (95% CI) during lockdown	3.4 (2.2–4.8)	2.9 (1.9–3.8)	3.5 (2.5–4.6)	2.9 (1.9–3.9)	3.4 (1.8–5.0)	3.4 (2.3–4.6)	2.8 (1.4–4.1)	3.0 (2.1–4.0)	3.7 (2.5–5.1)
	*p*-value	0.0974	0.7111	0.2636	0.3248	0.0335 *	0.2835	0.577	0.5119	0.2355
	Interaction *p*-value	0.17		0.67		0.0032 **			0.23	
Meat. Fish and Egg	Mean (95% CI) before lockdown	18.0 (16.0–19.9)	19.4 (14.5–21.4)	20.0 (17.9- 22.3)	17.7 (15.7–19.7)	18.9 (16.0–21.9)	18.9 (16.3–24.6)	18.2 (15.6–20.7)	19.5 (15.6–21.3)	16.9 (13.8–20.0)
	Mean (95% CI) during lockdown	17.8 (15.1–19.9)	19.1 (17.2–21.0)	19.9 (17.7–22.2)	17.3 (15.2–19.4)	19.8 (16.0–23.6)	19.0 (16.6–24.5)	16.8 (14.1–19.5)	18.9 (17.1–20.8)	17.3 (13.5–21.0)
	*p*-value	0.7255	0.5203	0.8217	0.4622	0.2625	0.8567	0.0388 *	0.2704	0.6352
	Interaction *p*-value	0.82		0.94		0.022 **			0.31	
Cereal	Mean (95% CI) before lockdown	17.7 (12.3–23.2)	18.6 (14.2–23.1)	17.8 (12.5.−23.1)	20.0 (14.4–25.6)	21.8 (108–32.8)	16.9 (12.1–21.8)	17.2 (11.6–22.7)	16.1 (12.5–20.0)	20.8 (13.3–28.3)
	Mean (95% CI) during lockdown	19.7 (13.8–25.7)	17.0 (13.0–21.1)	18.5 (12.3–24.5)	19.3 (14.2–24.4)	23.7 (11.3–36.2)	17.1 (12.9–21.4)	15.4 (10.4–20.4)	15.8 (12.6–19.0)	21.9 (13.2–30.5)
	*p*-value	0.0585	0.2806	0.5591	0.6729	0.3852	0.895	0.3906	0.7338	0.7126
	Interaction *p*-value	0.049 *		0.66		0.45			0.55	

Data is shown as the means of food intakes and their respective 95% confidence intervals (95% CI). Comparison between groups were performed using a paired two-tail Student’s *t*-test. Interaction *p*-value was calculated using a two-way ANOVA test to evaluate the effect of gender, age, body mass index (BMI) and capillary glycated haemoglobin (HbA1c) variables on the average weekly intake and exercise data, both before and during the COVID-19 lockdown. * *p* < 0.05, ** *p* < 0.005, *** *p* < 0.001, **** *p* < 0.0001.

**Table 3:** Comparison of physical activity before and during lockdown among patients with Type II Diabetes.

		Gender (*n* = 72)		Age (Years) (*n* = 72)		Body-Mass-Index (kg/m^2^) (*n* = 63)			Capillary HbA1c (*n* = 59)	
		Male	Female	44 to 63	64 to 77	25.0 to <30	30 to <35	35 to <40	<6.5%	≥6.5%
Moderate physical activity	Mean (95% CI) before lockdown	60.5 (30.9–90.1)	44.5 (26.4–62.5)	190.6 (70.4–310.8)	68.2 (15.0–121.5)	81.3 (−39.1–201.8)	102.9 (44.9–161.0)	124.0 (15.6–232.4)	125.7 (55.6–195.8)	57.2 (4.1–110.3)
	Mean (95% CI) during lockdown	20.6 (8.5–35.3)	21.2 (3.9–38.6)	33.8 (6.9–60.7)	48.4 (13.2–83.6)	32.7 (−6.4–71.8)	60.9 (18.9–102.9)	26.3 (−9.0–61.5)	43.6 (15.6–71.6)	40.3 (−10.6–91.1)
	*t*-test *p*-value	0.0007 ***	0.0005 ***	0.0119 *	0.3694	0.2803	0.0658	0.0933	0.0118 *	0.5591
	Interaction *p*-value	0.13		0.027 *		0.56			0.2	
Walking (min/week)	Mean (95% CI) before lockdown	363.2 (178.5–547.9)	268.4 (140.1–396.)	231.9 (147–316.8)	339.9	434.7 (195.2–647.1)	270.4 (110.6–430.3)	214.8 (119.2–3.10).	291.3 (165.8–416.8)	289.5 (124.0–455.0)
	Mean (95% CI) during lockdown	108.8 (37.6–180)	128.3 (16.5–240.1)	89.7 (16.2–163.2)	124.8	138.0 (−24.3–300.3)	113.3 (−19.8–246.3)	95.9 (25.0–166.7)	127.2 (26.28–228.1)	85.4 (−12.9–183.7)
	*t*-test *p*-value	0.0006 ***	0.0005 ***	0.0007 ***	<0.0001 ****	0.0037 **	0.0002 ***	0.0142 *	0.0003 ***	0.0004 ***
	Interaction *p*-value	0.14		0.077 *		0.13			0.56	
Sitting (hours/week)	Mean (95% CI) before lockdown	5.2 (4.6–5.6)	5.6 (4.8–6.3)	5.9 (5.1–6.7)	5.2 (4.5–5.9)	6.0 (5.0–7.0)	5.2 (4.3–6.1)	5.4 (4.5–6.2)	5.4 (4.7–6.0)	5.0 (4.2–5.9)
	Mean (95% CI) during lockdown	7.7 (6.9–8.5)3	6.8 (5.6–8.0)	7.3 (6.0–8.6)	7.3 (6.4–8.2)	7.5 (5.9–9.0)	6.7 (5.7–7.7)	7.2 (6.1–8.1).	6.9 (6.2–7.6)	7.2 (6.0–8.5)
	*t*-test *p*-value	<0.0001 ****	0.0213*	0.0332 *	<0.0001 ****	0.0516	0.001 **	0.0043**	<0.0001 ****	0.0002 ***
	Interaction *p*-value	0.07		0.35		0.52			0.25	

Data is shown as mean weekly physical activity and its respective 95% confidence intervals (95% CI). Comparison between groups were performed using a paired two-tail Student’s *t*-test. Interaction *p*-value was calculated using a two-way ANOVA test to evaluate the influence on dietary patterns. * *p* < 0.05, ** *p* < 0.005, *** *p* < 0.001, **** *p* < 0.0001.

**Table 4:** Correlation between FCQ-T and FCQ-S scores and dietary patterns, physical activity status and general characteristics of the sample. FCQ-S, Food Craving Questionnaire-State; FCQ-T, Food Craving Questionnaire-Trait.

Variables	FCQ-T		FCQ-S	
	Correlation	*p*-Value	Correlation	*p*-Value
Gender	0.227	0.018	−0.207	0.040
Age	−0.110	0.193	−0.069	0.292
Body-mass-index (kg/m^2^)	0.229	0.035	−0.284	0.012
Capillary HbA1c	0.176	0.091	0.265	0.021
Sugary food intake	0.093	0.220	0.082	0.248
Snacks food intake	0.227	0.029	0.163	0.089
SSB intake	0.149	0.110	0.150	0.108
Diary intake	−0.221	0.032	−0.221	0.032
Legume intake	−0.176	0.073	−0.153	0.103
Vegetable intake	0.308	0.005	0.417	0.0001
Fruit intake	0.210	0.039	−0.029	0.406
Nut intake	0.043	0.362	−0.027	0.413
Meat, fish and egg intake	−0.142	0.120	−0.222	0.032
Cereal intake	0.000	0.500	0.460	0.012
Moderate activity	0.132	0.136	0.180	0.066
Walking time	0.109	0.184	0.203	0.044
Sitting time	−0.085	0.242	−0.166	0.085

Data is shown as correlation (r Pearson) and *p*-value. Pearson correlation test was used. * *p* < 0.05.

## Supplementary Materials

The following are available online at https://www.mdpi.com/2072-6643/12/8/2327/s1, Table S1: Food intake frequencies to compare dietary patterns before and during the COVID-19 lockdown, expressed by percentage of patients according to weekly or daily frequency of intake of each food group. *n* = 72.
