# Supplementary material for: COVID-19 Lockdown and Changes of the Dietary Pattern and Physical Activity Habits in a Cohort of Patients with Type 2 Diabetes Mellitus
Source: Nutrients. 2020 Aug 4;12(8):2327. doi: 10.3390/nu12082327 (PMC7468739; doi:10.3390/nu12082327)
Supplement: Supplementary file 1 [file nutrients-12-02327-s001.pdf]

Table S1. Food intake frequencies to compare dietary patterns before and during COVID-19 lockdown, expressed by percentage of patients according to weekly or daily frequency of intake of each food group. *n*=72.

	Patients (%)	
	**BEFORE**	**DURING**
**Fruit frequency of intake per day**		
1 serving or less	55.7	51.4
2-4 servings	24.3	28,6
5 or more servings	20.,0	20,0
**Meat, fish and egg frequency of intake per day**		
1 serving or less	20,0	20.0
2 servings	47.1	48.6
3 or more servings	32.9	3.,4
**Cereal frequency of intake per day**		
1 serving or less	48.6	48.6
2-4 servings	41.4	42.,9
5 or more servings	10.0	8.6
**Nut frequency of intake per week**		
Never	22.9	28.6
1-3 servings	44.,3	32.,9
4 or more servings	32.,9	38.6
**Legumes frequency of intake per week**		
Never	12.9	11.4
1-3 servings	81.4	78.,6
4 or more servings	5.7	10.,0
**Sugar-sweetened beverage intake per week**		
Never	57.1	57.,1
1-3 servings	21.4	21.,4
4 or more servings	21.4	21.4
